# Aggregation of α-synuclein splice isoforms through a phase separation pathway

**DOI:** 10.1126/sciadv.adq5396

**Published:** 2025-04-16

**Authors:** Alexander Röntgen, Zenon Toprakcioglu, Samuel T. Dada, Owen M. Morris, Tuomas P. J. Knowles, Michele Vendruscolo

**Affiliations:** Centre for Misfolding Diseases, Yusuf Hamied Department of Chemistry, University of Cambridge, Cambridge CB2 1EW, UK.

## Abstract

The aggregation of α-synuclein (αSyn) is associated with Parkinson’s disease and other related synucleinopathies. Considerable efforts have thus been directed at understanding this process. However, the recently discovered condensation pathway, which involves the formation of phase-separated liquid intermediate states, has added further complexity. In parallel, it has been reported that different αSyn splice isoforms may be implicated in aggregate formation in disease. In this study, we compare the phase behavior of four αSyn isoforms (αSyn-140, αSyn-126, αSyn-112, and αSyn-98). Using different biophysical tools including confocal microscopy, kinetic assays and microfluidic-based approaches, we find stark differences between the four systems in their propensities to undergo phase separation and aggregation. Furthermore, we show that even small amounts of αSyn-112, one of the predominant isoforms after αSyn-140, can affect the phase separation of αSyn-140. These results highlight the importance of conducting further investigations to elucidate the role of alternative splicing in synucleinopathies.

## Teaser

A study reveals that splice isoforms of α-synuclein can alter the condensation pathway of this protein.

## INTRODUCTION

The self-assembly of α-synuclein (αSyn) into amyloid fibrils is the hallmark of a family of neurodegenerative diseases termed synucleinopathies, which include Parkinson’s disease (PD), dementia with Lewy bodies, and multiple system atrophy (*1*–*7*). PD alone is estimated to affect over 6 million people worldwide, causing prominent motor symptoms and often cognitive impairment (*2*–*7*), with no disease-modifying treatments currently available (*8*).

Given the central role of αSyn in synucleinopathies, its aggregation process has been subject of extensive research efforts. The amino acid sequence of αSyn is encoded by the *SNCA* gene and divided into three functional regions—an amphipathic N terminus (residues 1 to 60), a hydrophobic non–amyloid-β component (NAC) region (residues 61 to 95), and a flexible, negatively charged C terminus (residues 96 to 140) (*9*). Multiple studies have focused on the aggregation of αSyn through the so-called deposition pathway, which describes aggregation into amyloid fibrils directly from native monomers (*10*–*12*). In this context, the NAC region and C terminus of αSyn have been described as promoting and inhibiting aggregation, respectively (*13*, *14*).

Moreover, increasing evidence suggests that αSyn does not only exist as a single protein of 140 amino acids (αSyn-140) but rather as a mixture of various related proteoforms (*15*–*18*). Similar to many proteins, the diversity of αSyn species is greatly enhanced by posttranslational modifications at the protein level (*19*–*21*) and alternative splicing at the transcript level (*22*–*28*). Besides the full-length αSyn-140 variant, other splice isoforms, including αSyn-126, αSyn-112, and αSyn-98, are generated via alternative splicing of the *SNCA* transcript, by skipping exon 3 (amino acids 41 to 54), exon 5 (amino acids 103 to 130), or both, respectively (*22*–*26*). Because of their differences in sequence composition, these four splice isoforms of αSyn exhibit different biophysical properties. We previously showed that αSyn-112 and αSyn-98 are less soluble and exhibit enhanced aggregation kinetics while forming morphologically distinct aggregates in the deposition pathway (*29*).

Further complexity in the study of αSyn aggregation comes from the recent observation that liquid-liquid phase separation (LLPS; referred to as condensation in the following) underlies an additional pathway for biomolecular self-assembly, leading to the formation of dense liquid-like condensates from initially dilute solutions. Notably, condensates of αSyn (*30*) and other amyloidogenic proteins such as tau (*31*), amyloid-β (*32*), fused in sarcoma (FUS) (*33*–*36*), and transactive response DNA binding protein 43 (TDP-43) (*37*) can further undergo a liquid-to-solid transition to form amyloid aggregates. This type of aggregation, which is dependent on the prior formation of a dense liquid-like intermediate state, has been termed the condensation pathway.

Since initial reports of αSyn-140 undergoing condensation, several studies have examined the effect of external modulators (*30*, *38*–*40*). For example, addition of salts or hexanediol, which disrupt electrostatic and hydrophobic interactions, respectively, has shown that both types of interactions may be involved in αSyn condensation and aggregation within condensates (*30*). Metal ions have also been reported to affect αSyn condensation and aggregation within condensates (*41*–*43*). Lipid vesicles, alone or in synergy with metal ions, may also promote these processes (*39*, *42*). Recent work has demonstrated that pharmacological modulation by small organic molecules or peptides can alter αSyn condensation and aggregation within condensates (*44*–*46*). A variety of techniques have been used to study phase separation and aggregation including fluorescence, spectroscopy, electron microscopy, or complementary methods such as centrifugation or scattering-based techniques (*30*, *45*, *47*, *48*).

In this study, we assessed the effects of physiologically occurring exon deletions on the condensation and subsequent aggregation behavior of αSyn. By using confocal microscopy, we compared the phase boundaries using a previously established drop-casting assay (*30*) to obtain an estimate of the condensation propensity of the different αSyn splice isoforms. Furthermore, we visualized condensate formation and measured the aggregation within condensates in real time. In addition, we used a microfluidic-based technique to determine the critical concentrations for condensation and aggregation within the condensates.

## RESULTS

**Fig. 1.**
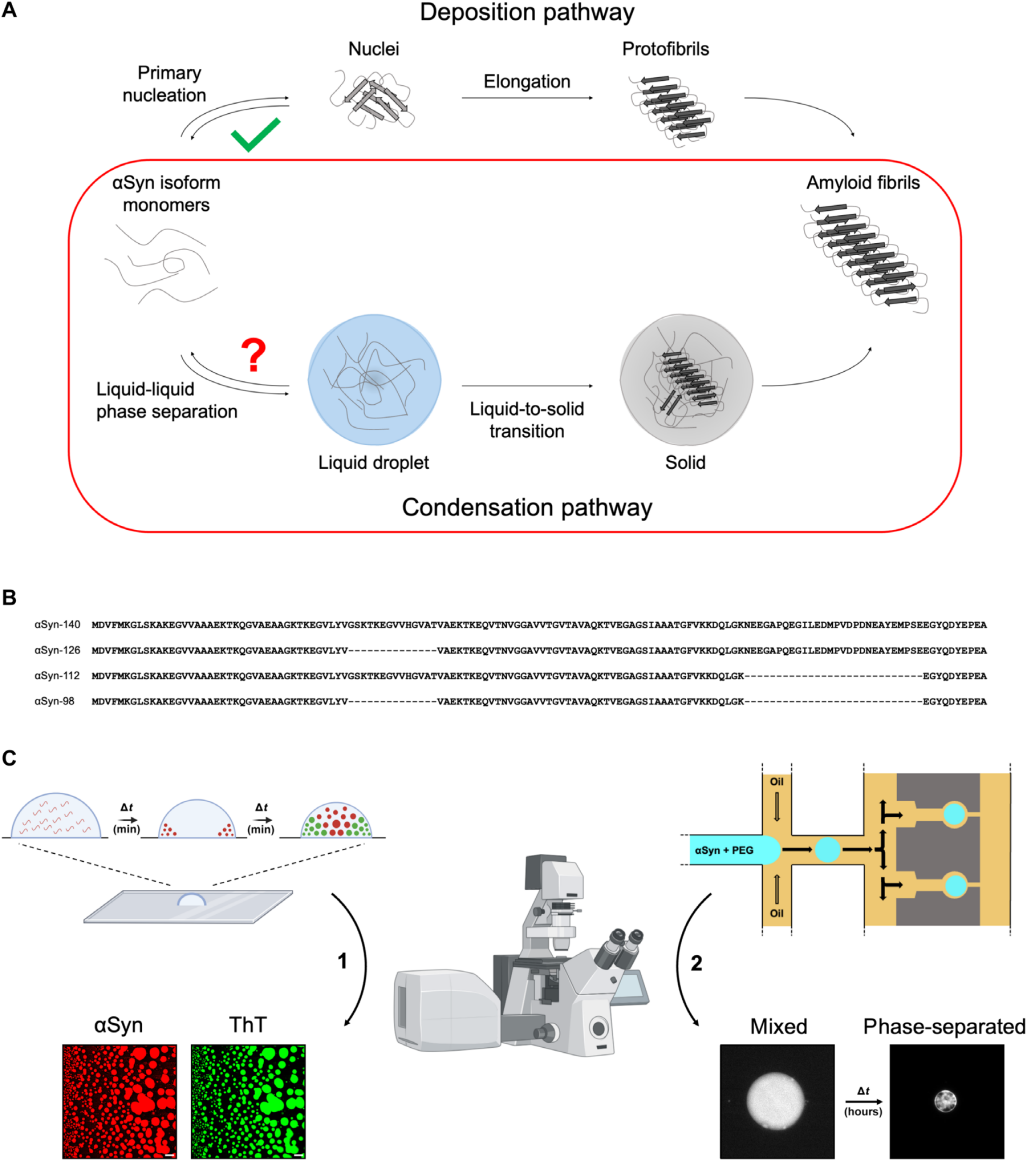
Schematic overview of the condensation of αSyn splice isoforms studied in this work. (**A**) αSyn isoforms can assemble into amyloid aggregates following the deposition and condensation pathways. While the deposition pathway of these splice isoforms has been recently described (*29*), the condensation pathway was investigated in this study. (**B**) Comparison of the amino acid sequences of the αSyn splice isoforms. αSyn-140 contains the full amino acid sequence, and αSyn-126 and αSyn-98 lack residues 41 to 54 located in the amphipathic N terminus, whereas αSyn-112 and αSyn-98 lack residues 103 to 130 located in the negatively charged, flexible C terminus. (**C**) Experimental approaches used in this study. Drop-casting assay (left): A mixture of αSyn splice isoform (1% fluorescent label) and polyethylene glycol (PEG) is spotted on a glass dish and monitored over time by confocal microscopy. Amyloid aggregation is visualized by the addition of the amyloid-binding dye thioflavin T (ThT) (*30*). A deposited drop evaporates over time, increasing the concentrations of its components and inducing condensation and subsequent aggregation. Microfluidics (right): Water-in-oil droplets of αSyn and PEG are generated in an oil phase and trapped in the device. Through water-in-oil droplet shrinkage, we can observe condensation and obtain biophysical parameters such as the critical concentration needed to induce condensation or aggregation. Elements of this figure have been adapted using BioRender.com.

In this study, we used a combination of biophysical methods to investigate the condensation and the aggregation within condensates of four αSyn splice isoforms (αSyn-140, αSyn-126, αSyn-112, and αSyn-98). The two main pathways by which αSyn can transition from native monomers to amyloid fibrils are schematically illustrated in Fig. 1A. Here, we investigated the previously uncharacterized condensation pathway of the αSyn splice isoforms (shown in the red box in Fig. 1A). The locations of exon deletions between these αSyn splice isoforms are shown in Fig. 1B. To assess the time-dependent formation of αSyn condensates, we built on a previously established drop-casting method (*30*), allowing us to monitor condensate formation using confocal microscopy (Fig. 1C, left). Furthermore, we used a microfluidic-based approach together with confocal microscopy to derive the critical concentrations needed for condensation and for aggregation within condensates to occur (Fig. 1C, right) (*49*, *50*).

### Phase boundaries of αSyn splice isoforms vary with exon deletions

**Fig. 2.**
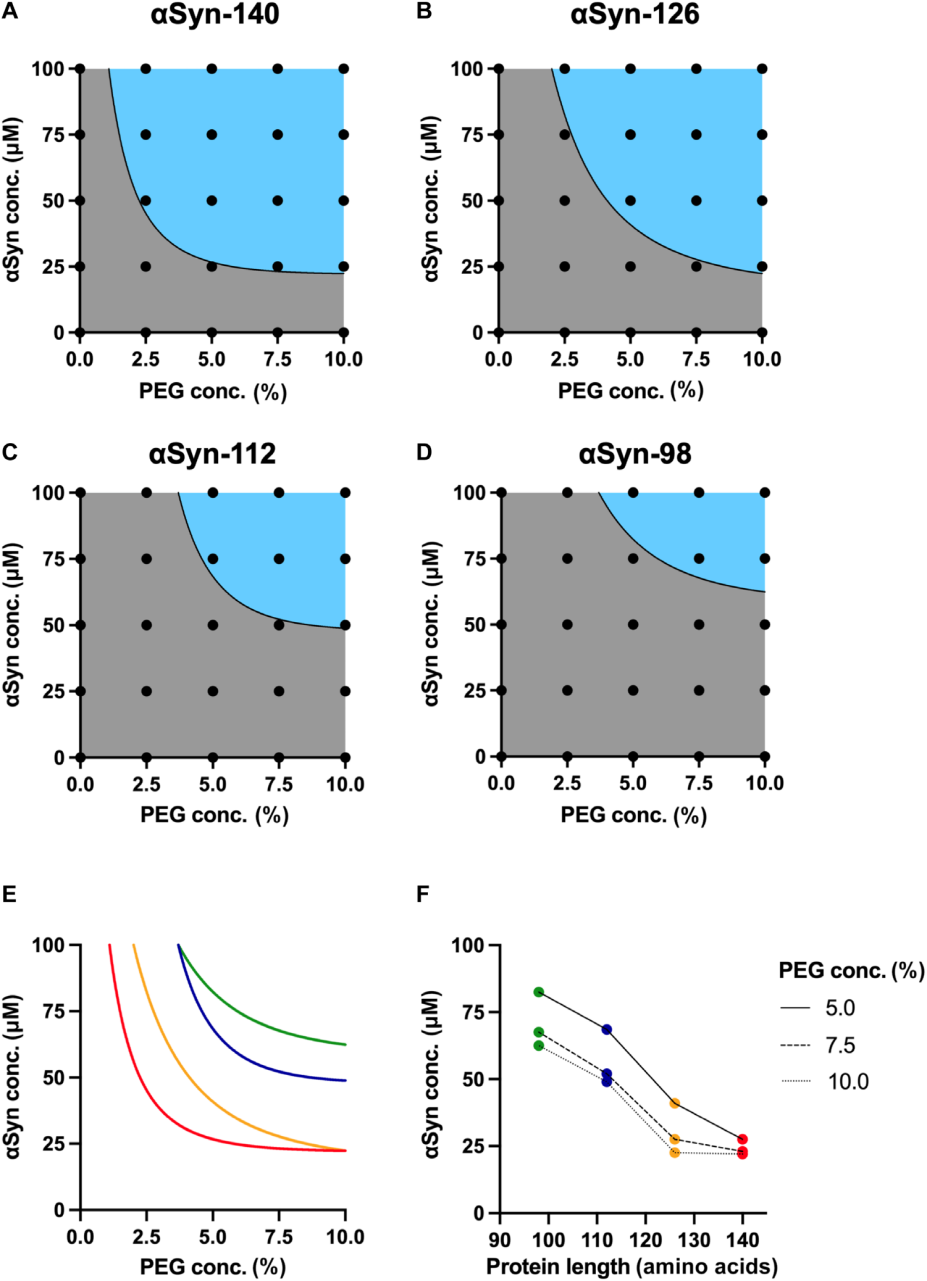
Phase diagrams of condensation of the four αSyn splice isoforms. (**A** to **D**) Phase diagrams of condensation of (A) αSyn-140, (B) αSyn-126, (C) αSyn-112, and (D) αSyn-98 were constructed using the drop-casting method, i.e., by depositing drops containing different αSyn and PEG concentrations for 10 min on a glass slide. The black dots represent the conditions investigated. The mixed and demixed phases are shown in gray and blue, respectively. The black line was added as a visual guidance for the phase boundary. (**E**) Comparison of the phase boundaries for condensation of αSyn-140 (red), αSyn-126 (orange), αSyn-112 (blue), and αSyn-98 (green), which reveals a shift to higher initial αSyn and PEG concentrations required for condensation. (**F**) Initial αSyn concentrations required for condensation, which were estimated on the basis of the phase boundaries for condensation at fixed PEG concentrations, reveal a decreasing trend with increasing sequence length.

To assess the propensity of the αSyn splice isoforms to undergo condensation, we first determined their phase boundaries. For this, drops of a solution containing αSyn and polyethylene glycol (PEG), which acts as a molecular crowding agent known to induce condensation (*30*, *40*, *45*), were pipetted on a glass surface and monitored over time using confocal microscopy. By systematically varying the initial αSyn and PEG concentrations, we compared the condensation propensity of the different αSyn splice isoforms at a fixed time point (10 min) after drop casting. A sample was considered phase-separated if condensates were observed in all replicates (Fig. 2). Representative confocal microscopy images of the phase boundary conditions are shown in fig. S1. αSyn-140 was found to be most prone to undergoing condensation compared to the other splice isoforms. This was followed by αSyn-126, αSyn-112, and, lastly, αSyn-98 (Fig. 2, A to E). Moreover, this effect was further highlighted by estimating the initial αSyn concentration required for condensation at fixed PEG concentrations, which shows a decreasing trend with increasing protein length (Fig. 2F).

### Different condensate sizes and numbers of the αSyn splice isoforms

**Fig. 3.**
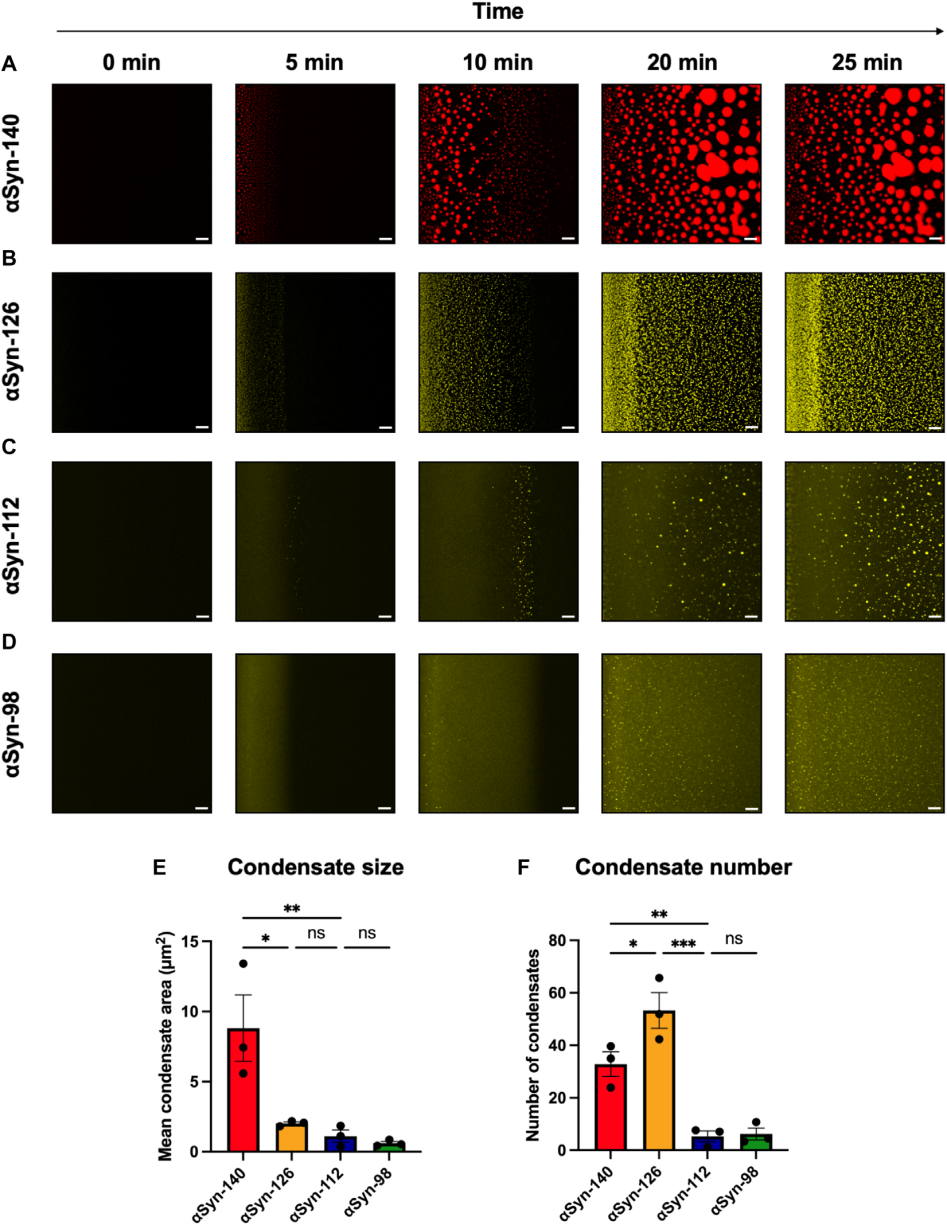
Time evolution of the condensates of αSyn splice isoforms. (**A** to **D**) Fluorescence images showing the condensate formation of αSyn splice isoforms. For this, a drop containing 100 μM αSyn and 10% PEG was deposited on a glass surface and monitored using confocal microscopy. αSyn condensates were visualized by addition of 1% fluorophore-labeled αSyn. Scale bars, 20 μm. (**E** and **F**) Quantification of (E) the size and (F) number of condensates per 1000 µm^2^, observed at 20 min. Data are shown as means ± SEM of independent experiments (*n* = 3). One-way analysis of variance (ANOVA) with Tukey’s post hoc test, ***P < 0.001, **P < 0.01, and *P < 0.05. ns, nonsignificant.

We next evaluated the time evolution of αSyn condensates. The condensates start forming at the edge of the drop, grow, and move toward the center of the drop. Subsequently, consistent with recent results, αSyn-140 readily undergoes amyloid aggregation within condensates (*30*). To probe this process, we chose conditions where all four isoforms are prone to undergoing condensation (100 μM of different αSyn isoforms and 10% PEG; Fig. 2). Condensates were visualized by adding a small amount (1%) of fluorophore-labeled αSyn (Fig. 3A to D), thus ensuring that any influence from the dye was negligible. The cysteine mutant of αSyn-140 was labeled with Alexa Fluor 647 (red), whereas the cysteine mutants of αSyn-126, αSyn-112, and αSyn-98 were labeled with Alexa Fluor 555 (yellow). For αSyn-140 and αSyn-126, spherical condensates can be observed early at the edge of the drop at 5 min (Fig. 3, A and B). However, for αSyn-112 and αSyn-98, a continuous area of fluorescence appears at first (Fig. 3, C and D), which may be due to the appearance of a large number of small condensates below the resolution limit of the microscope. In contrast, spherical condensates are only observed toward the center of the drop at later time points (Fig. 3, C and D). Furthermore, we observed that αSyn-140 formed significantly larger condensates than the other three isoforms (Fig. 3E), while αSyn-126 formed the greatest number of condensates (Fig. 3F). Both αSyn-140 and αSyn-126 formed significantly more condensates than αSyn-112 and αSyn-98 (Fig. 3F), which is indicative of the increased propensity of αSyn-140 and αSyn-126 to phase separate. However, it should be noted that, if small condensates below the resolution are present, then they would increase the effective number of condensates. Together, the results indicate a slower progression of condensate formation for shorter isoforms, as reflected by the size and number of condensates.

### Determining the critical concentration at which αSyn splice isoforms phase separate

**Fig. 4.**
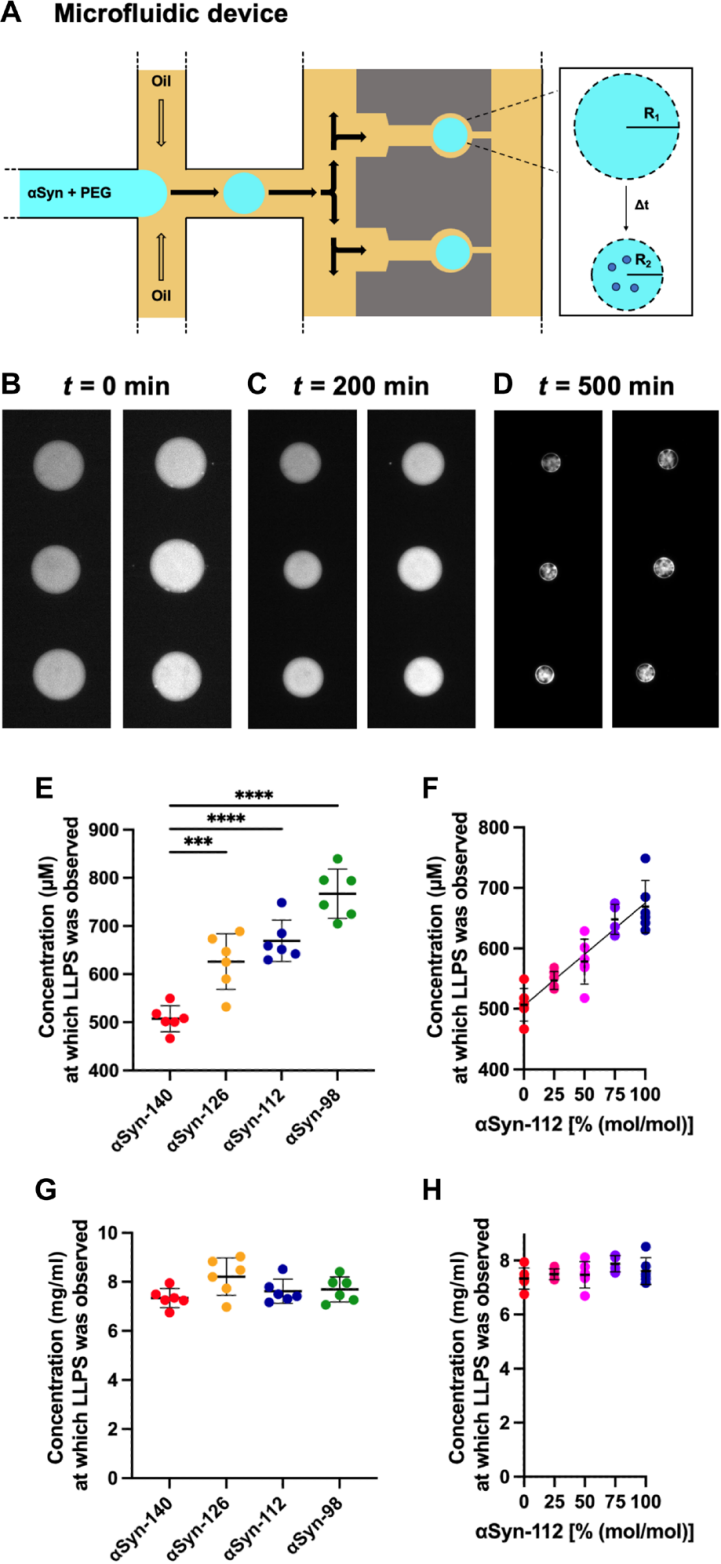
Determination of the critical concentration for condensation of αSyn splice isoforms using microfluidics. (**A**) Schematic of the microfluidic device used. Water-in-oil droplets containing αSyn and PEG are generated using a stream of fluorinated oil and subsequently trapped in the device. The water-in-oil droplets shrink over time in a controlled manner, increasing the αSyn concentration within the water-in-oil droplets. (**B** to **D**) Representative images showing the shrinkage of water-in-oil droplets and formation of condensates of αSyn-126 in the microfluidic device. For all splice isoforms, 100 μM αSyn (1% fluorescent label) and 10% PEG were used as initial concentrations. (**E**) The critical concentration for condensation shows a significant increase for the αSyn splice isoforms with decreasing sequence length. One-way ANOVA with Tukey’s post hoc test, ****P < 0.0001 and ***P < 0.001. (**F**) Critical concentration of a mixture of αSyn-140 and αSyn-112 increases with the relative content of αSyn-112. Data are shown as means ± SD of different droplets (*n* = 6). (**G** and **H**) When measured in terms of mass concentration, rather than molar concentration [as in (E) and (F)], the critical concentrations for condensation do not depend on the identity of the αSyn splice isoforms.

To further corroborate our findings, we used a microfluidic device that generates and traps water-in-oil droplets containing the aqueous αSyn solution (*49*, *50*). The water-in-oil droplets shrink in a controlled manner over the course of several hours, increasing the concentration of αSyn inside the water-in-oil droplets and, thus, inducing condensation (*30*, *32*, *45*). Comparing the radii of the water-in-oil droplets at the beginning of the experiment (i.e., at the initial state of the protein) and at the point of condensate formation, therefore, allows us to calculate the critical αSyn concentration required for condensation (Fig. 4, A to D). Using this approach, we show that the critical concentrations for condensation exhibit significant differences between αSyn splice isoforms. The αSyn concentration at the start of the experiment was set to 100 μM for all four splice isoforms, containing 1% fluorophore-labeled αSyn for detection of condensates by confocal microscopy. Following water-in-oil droplet shrinkage, condensation was induced and eventually observed for αSyn-140 at a concentration of 507 ± 27 μM (averaged over six droplets). The critical concentration required for condensation increased as a function of decreasing isoform size. Condensation was observed at a concentration of 626 ± 58 µM for αSyn-126, 669 ± 43 μM for αSyn-112 and 767 ± 51 μM for αSyn-98 (Fig. 4E). This constitutes a 1.5-fold difference between αSyn-140 and αSyn-98.

Next, we assessed the effect of an alternative splice variant on the phase behavior of αSyn-140. To probe this, we chose αSyn-112 as we previously found that this isoform modulates the aggregation of αSyn-140 in the deposition pathway (*29*). We therefore assessed the critical concentrations required for condensation of mixtures containing varying ratios of these two isoforms (Fig. 4F). We found a linear correlation between the content of αSyn-112 and the critical concentration for condensation, confirming that the presence, even at relatively low concentrations, of alternative splice isoforms may influence the phase behavior of αSyn-140. Moreover, we confirmed that mixing αSyn-140 with its alternative isoforms leads to the formation of cocondensates rather than distinct condensates for each isoform (fig. S2). In every mixture, the alternative isoform was immediately recruited into condensates with αSyn-140. This supports the idea that the alternative splice isoforms may interact with αSyn-140 in the liquid phase and, therefore, influence its condensation behavior. We also found that when measured in terms of mass per volume (Fig. 4, G and H), rather than number of molecules per volume (Fig. 4, E and F), the critical concentrations for condensation are independent from the identity of the αSyn splice isoforms.

### Condensation and deposition pathways of aggregation of αSyn splice isoforms

In a similar fashion to investigating the condensation of the αSyn splice isoforms, we used confocal microscopy to monitor their amyloid aggregation by measuring thioflavin T (ThT) fluorescence. Upon binding to amyloid fibrils with β sheet structure, the quantum yield of this fluorophore increases significantly, and, thus, one can correlate the presence of aggregates with the fluorescence signal. This renders ThT a good reporter dye of amyloid aggregation and is widely used to probe protein aggregation (*51*).

**Fig. 5.**
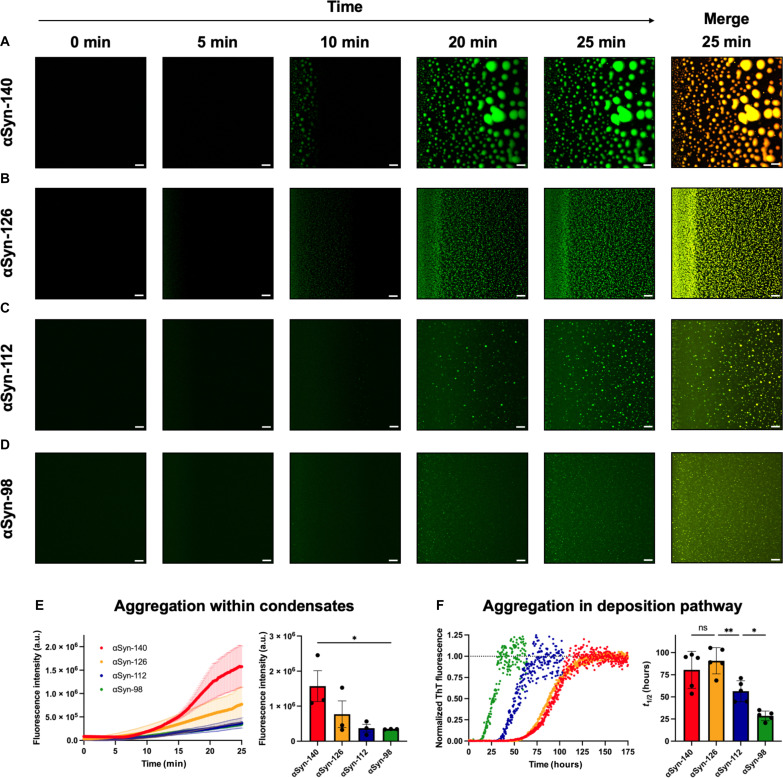
Aggregation kinetics of αSyn splice isoforms within condensates. (**A** to **D**) Fluorescent images showing amyloid aggregation within condensates for the four splice isoforms. For this, a drop containing 100 μM αSyn, 10% PEG, and 20 µM ThT was deposited on a glass surface and monitored using confocal microscopy. Amyloid aggregation was monitored in the same drop as in Fig. 3. Merged images of Alexa Fluor channels (condensates) and ThT channels (amyloid aggregates) at 25 min are shown as the right panels. Merged images at all time points are shown in fig. S3. Scale bars, 20 μm. (**E**) ThT fluorescence intensity over time (left) and plateau value (right). Data are shown as means ± SEM (*n* = 3). One-way ANOVA with Dunnett’s post hoc test, *P < 0.05. a.u., arbitrary units. (**F**) Normalized aggregation traces in the deposition pathway using plate reader–based time-resolved fluorescence measurements (left) and the corresponding aggregation half-times (right). Data are shown as means ± SD. One-way ANOVA with Tukey’s post hoc test, **P < 0.01 and *P < 0.05. ns, nonsignificant.

ThT fluorescence, using confocal microscopy, was monitored in parallel to observing condensate formation (Fig. 5). ThT-positive condensates were formed by all four αSyn splice isoforms, confirming their capacity to undergo amyloid aggregation following condensation (Fig. 5, A to D). For αSyn-140 and αSyn-126, ThT-positive condensates were observed at 10 min (Fig. 5, A and B), although much less pronounced for αSyn-126 (Fig. 5B). For αSyn-112 (Fig. 5C) and αSyn-98 (Fig. 5D), ThT-positive condensates were observed at 20 min, which is in line with their decreased propensity to undergo condensation and subsequent aggregation. Moreover, especially in the case of αSyn-98, a continuous area of ThT fluorescence is formed (Fig. 5D). This finding further supports our prior hypothesis (Fig. 3) that this area is populated by condensates below the resolution limit. However, it is clear from the ThT fluorescence signal that aggregation within these condensates may still occur. We quantified the change in ThT fluorescence over time, revealing a decreasing trend of final ThT fluorescence intensity with decreasing protein length (Fig. 5E). For all cases other than αSyn-140, the traces did not reach the plateau phase before drying of the system occurred, prohibiting further normalization and kinetic analysis. However, our results indicate a lower propensity to aggregate in the condensation pathway for shorter αSyn splice isoforms. This is opposite to findings for the deposition pathway of aggregation using a classical plate reader–based aggregation assay (Fig. 5F), showing that the shorter isoforms αSyn-112 and αSyn-98, which lack the sequence encoded by exon 5, aggregate significantly faster. Therefore, contrasting trends seem to underlie the aggregation propensity in these two different pathways.

**Fig. 6.**
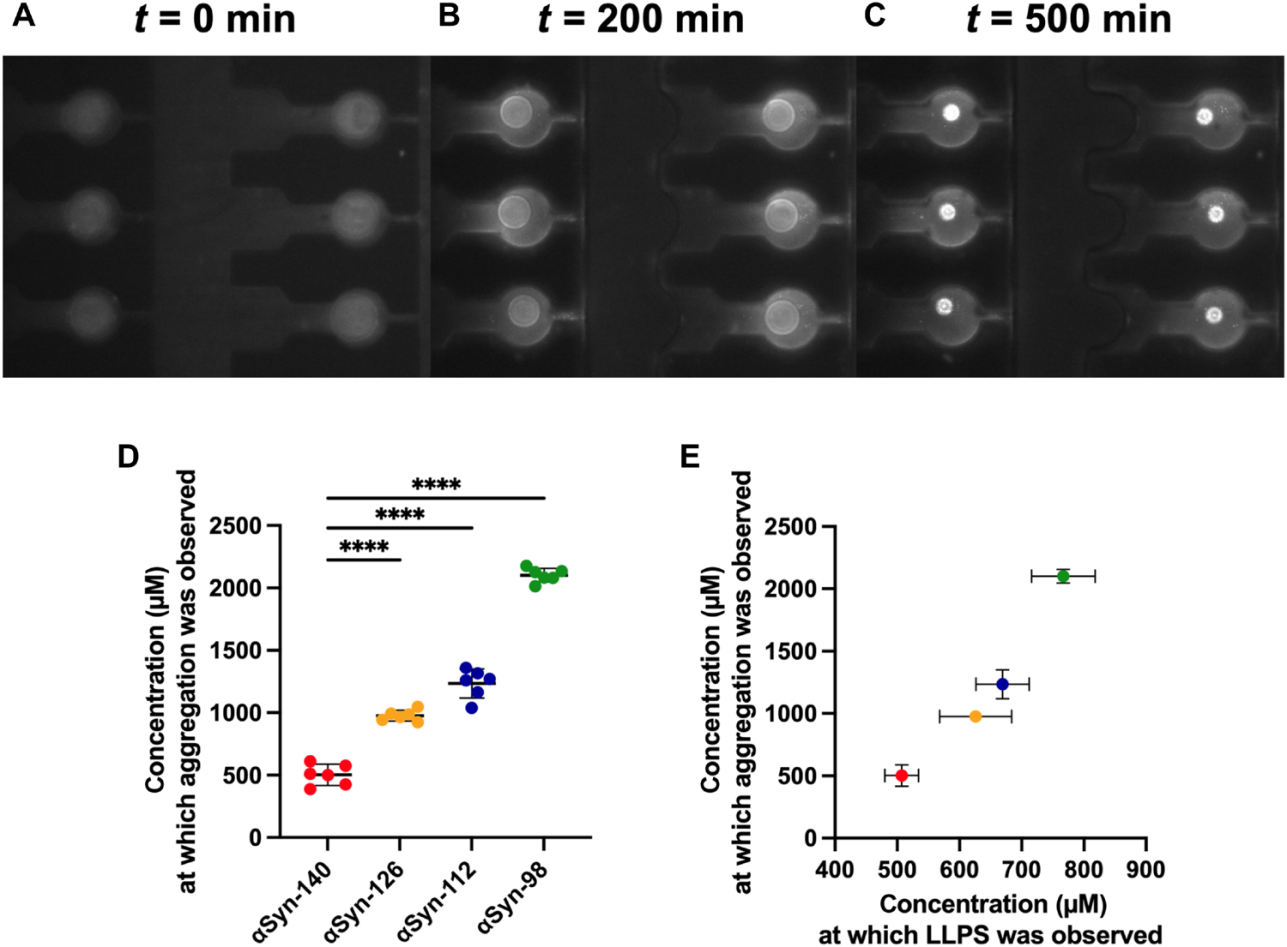
Determination of the critical concentration for aggregation of αSyn splice isoforms using microfluidics. (**A** to **C**) Representative images showing the shrinkage of water-in-oil droplets and the formation of ThT-positive aggregates of αSyn-126. For all splice isoforms, 100 μM αSyn, 10% PEG, and 20 μM ThT were used as starting concentrations. (**D**) Critical concentration of αSyn for aggregate formation within condensates. Data are shown as means ± SD of different water-in-oil droplets (*n* = 6). One-way ANOVA with Tukey’s post hoc test, ****P < 0.0001. (**E**) Comparison of the critical concentrations for condensation and for aggregation of the splice isoforms of αSyn (red, αSyn-140; orange, αSyn-126; blue, αSyn-112; green, αSyn-98).

Last, we determined the critical concentration of αSyn required for aggregation within condensates using a microfluidic device (Fig. 6). As in the previous microfluidic experiment to determine the critical concentration for condensation (Fig. 4), the starting concentration of all αSyn splice isoforms was set to 100 μM. To avoid any cross-talk between the fluorescently labeled αSyn and the fluorophore used to monitor the aggregation of αSyn, we only used wild-type αSyn. The mixture contained 20 μM ThT for the detection of amyloid structures. Hence, ThT intensity was monitored over time (Fig. 6, A to C), and the critical concentration for aggregation was calculated following shrinkage of the water-in-oil droplets. Notably, we observed an even stronger dependence of the critical αSyn concentration required for aggregation based on protein length (Figs. 4E and 6D), increasing by about a factor of 4 from αSyn-140 to αSyn-98. Aggregation was first observed at a concentration of 502 ± 85 μM for αSyn-140, which increased to 976 ± 43 μM for αSyn-126, 1234 ± 117 μM for αSyn-112, and 2102 ± 56 μM for αSyn-98 (Fig. 6D). Upon phase separation, αSyn-140 appeared to have the propensity to immediately aggregate through the condensation pathway. This is in stark contrast to the other splice isoforms, where a lag time existed between the point at which phase separation was initially observed, to the point at which the condensates started maturing and aggregating. While using the drop-casting method, we observed a slight delay between the condensation and the aggregation of αSyn-140. This can be attributed to the air-water interface influencing the protein phase space. This interface has been shown to influence the aggregation and phase space of other amyloidogenic proteins as well (*52*, *53*). However, with the microfluidic setup, we avoid such an interface and have an inert oil surrounding the aqueous droplet, rendering the microfluidic approach much more sensitive. Thus, shorter αSyn splice isoforms do require not only a higher absolute concentration to undergo aggregation within condensates but also a higher relative concentration compared to that of condensation. In addition, we used transmission electron microscopy to investigate the morphology of the aggregates formed within the condensates (fig. S4). For all splice isoforms, we observed that the formed aggregates were fibrillar (fig. S4), which is in line with the increase in fluorescence of ThT (Figs. 5 and 6) and as previously reported for αSyn-140 (*29*, *30*). We show that washing steps of the grid are required to remove residual PEG and visualize the fibrillar aggregates (figs. S4 and S5). Without doing these washing steps, no fibrillar aggregates could be observed (fig. S5). We further compared the amyloid formation of the four splice isoforms by Fourier transform infrared spectroscopy. All isoform aggregates have the characteristic cross–β sheet signature but exhibit individual profiles in their amide I and II bands (fig. S6). The slight shift of αSyn-112 and αSyn-98 toward lower wave numbers at the amide I peak (~1630 cm^−1^) implies that they have a larger β sheet content compared to αSyn-140 and αSyn-126.

## DISCUSSION

In this study, we have provided a detailed biophysical characterization of the aggregation behavior within condensates of three splice isoforms of αSyn (αSyn-126, αSyn-112, and αSyn-98) and compared it to that of the main isoform (αSyn-140). Our study has revealed that the propensity to undergo condensation and the propensity to aggregate within condensates are different for the distinct splice isoforms.

Because of its flexibility, the C terminus of αSyn has previously been described as inhibiting aggregation in the deposition pathway but has also been predicted to be a main driver of αSyn condensation (*38*). In our recent study about αSyn splice isoforms regarding the aggregation of these isoforms through the deposition pathway, we have demonstrated that deletion of the C-terminal exon 5, as in αSyn-112 and αSyn-98, accelerates the aggregation kinetics, while deletion of exon 3, as in αSyn-126 and αSyn-98, displayed a lesser effect on the aggregation kinetics (*29*).

Our findings could also be compared with the behavior of αSyn truncation variants as client or codriving proteins in phase-separating systems (*48*, *54*, *55*). The full-length variant αSyn-140, the C-terminally truncated variant αSyn-108, and part of the NAC region (amino acids 68 to 78) had varying propensities to be recruited into preexisting biomolecular condensates, which consisted of different (poly)cations and (poly)anions, with differing surface charges. Moreover, the tested variants aggregated with different kinetic rates in the presence of these condensates. Notably, while αSyn-140 was recruited into all three types of condensates, αSyn-108 was not recruited into any of the systems (*54*). Moreover, αSyn-132 and αSyn-102 compared to αSyn-140 were shown to have a decreasing propensity to form cocondensates with the prion protein (*55*). Together, these studies highlight the importance of the C terminus of αSyn in heterotypic condensate formation.

Here, we explored the role of four of the most prevalent αSyn splice isoforms to probe the effects of sequence variations on the condensation and aggregation behavior of αSyn. Using a drop-casting method and confocal microscopy, we determined a shift in the phase boundaries between the four isoforms according to protein length, as well as differences in condensate size, number, and ThT intensity (Figs. 2, 3, and 5). In addition, using a microfluidic approach, we calculated the two critical concentrations of αSyn required for condensation and for aggregation for each isoform (Figs. 4 and 6). The observed trend shows that the propensities for condensation and aggregation are not very sensitive to the exact composition and location of the deleted sequences, i.e., in the N terminus (exon 3) or C terminus (exon 5).

Similarly, in a recent study examining C-terminally truncated variants compared to αSyn-140, the condensation propensity decreased with progressing truncation in the presence of 50 mM NaCl, although this trend was reversed in the absence of additional salt (*48*). In another study investigating the effect of sequence complexity of αSyn by introducing sequence modifications, a correlation was reported between the propensities for condensation and fibrillation under LLPS conditions (*56*). However, different metrics were used to probe condensation and aggregation propensities, making it unclear whether this correlation is only a consequence of the fact that the reduced condensation also slows down subsequent aggregation. The authors showed that there is no correlation between fibrillation speed under non-LLPS conditions (using shaking) and condensation propensity between different sequence variants. This further supports our finding that the biophysical rules underlying αSyn deposition and condensation are uncoupled.

By analyzing both processes, condensation and aggregation, using the same microfluidic approach, we could calculate the critical concentrations and, thus, directly compare the two pathways for all four αSyn splice isoforms. We observed a decrease in aggregation propensity proportional to, but stronger than, that of the condensation propensity with decreasing protein length, as quantified by the αSyn critical concentrations. The results show that the propensities of αSyn for condensation and aggregation within condensates are not strongly affected by the biophysical properties of the deleted regions of the sequence, which vary strongly between exon 3 in the amphipathic N terminus and exon 5 in the negatively charged, flexible C terminus. This finding differs from recent results concerning the behavior of splice isoforms in the deposition pathway, where aggregation is strongly dependent on the sequence composition of the deleted exons (*29*). Thus, the length of the sequence appears to be a major determining factor for the phase separation propensity of the αSyn isoforms, at least under the conditions tested. This finding can be explained by the fact that longer proteins have greater entropy, which stabilizes the condensed state. Moreover, because of the increased length, the proteins can form weak, transient interactions, which have been shown to be crucial for phase separation, as previously described for other protein systems (*57*–*59*). As most previous studies of αSyn phase separation have involved the use of PEG (*40*, *42*, *43*, *48*, *56*, *60*), we too used this approach in our assay. We note that the weak dependence of the phase behavior on the protein sequence may be caused by the presence of PEG. These results highlight the importance of further studies aimed at finding physiological triggers of αSyn condensation.

We also note that increasing evidence indicates a change in the expression of αSyn isoforms in brain tissue or cell lines derived from patients suffering from synucleinopathies, including the down-regulation of some of the shorter isoforms (*61*–*64*). As we found these shorter isoforms to be less condensation prone in our study, these results support further investigations into their relevance in disease. According to this view, a loss of less condensation-prone isoforms may contribute to the pathogenesis of a certain subset of neurodegenerative diseases.

In conclusion, the findings that we reported extend our understanding of the phenomenon of αSyn condensation and subsequent aggregation within condensates, as well as the role of different splice variants in these processes. While the relative contributions of the condensation and deposition pathways in pathological processes remain to be elucidated, the biophysical rules that we have uncovered in this study may also apply for other proteins involved in other neurodegenerative diseases. Future studies should address the role of sequence variations in these processes to understand the effects of changes in the proteoform spectrum on neurodegenerative processes.

## METHODS

### Recombinant production and fluorophore labeling of αSyn splice isoforms

αSyn splice isoforms (αSyn-140, αSyn-126, αSyn-112, and αSyn-98) and their cysteine mutants A90C (αSyn-140-Cys and αSyn-112-Cys) and A76C (αSyn-126-Cys and αSyn-98-Cys) were expressed in BL21(DE3) bacteria and purified in 50 mM tris-HCl (pH 7.4), as described previously (*14*, *30*, *65*) (fig. S7). The mutated residues between the splice isoforms are identical, only the residue numbering differs because of the N-terminal deletions in αSyn-126 and αSyn-98. Protein stocks of these cysteine mutants were incubated with 1 mM dithiothreitol (DTT) for 30 min at room temperature (RT), and DTT was removed using Amicon Ultra-15 Centrifugal Filter Units 10 molecular weight cutoff (MWCO) (αSyn-140-Cys) or 3 MWCO (αSyn-126-Cys, αSyn-112-Cys, and αSyn-98-Cys). Cysteine mutants were then incubated with a 1.5-fold molar excess of Alexa Fluor 647 C2 maleimide (αSyn-140-Cys) or Alexa Fluor 555 C2 maleimide (αSyn-126-Cys, αSyn-112-Cys, and αSyn-98-Cys) and a 5-fold molar excess of tris(2-carboxyethyl)phosphine (TCEP) with constant mixing by rotation at 4°C overnight. Unbound dye and TCEP were then removed using Amicon Ultra-15 Centrifugal Filter Units, and the final concentration of labeled protein was measured by ultraviolet-visible spectroscopy using a Cary 100 system (Agilent). All proteins were snap frozen in liquid N_2_ and stored at −80°C until further use.

### Phase separation assay

αSyn splice isoforms were mixed with 1% of their fluorophore-labeled variants in 50 mM tris-HCl (pH 7.4), with PEG-10,000 (Thermo Fisher Scientific). PEG was prepared as a 50% (w/w) stock solution in 50 mM tris-HCl (pH 7.4), and PEG concentrations in this study are expressed as a volume concentration [% (v/v)] of this stock solution. For the determination of phase boundaries, protein and PEG concentrations were systematically varied. Five drops (10 μl) were deposited on Poly-Prep glass slides (Merck Millipore) and incubated at RT for 10 min. A condition was considered phase separated if condensates were visible in all replicates using a 10× objective on a Leica Stellaris 5 inverted confocal microscope. For time-resolved assessment of condensate and amyloid formation, αSyn splice isoforms (100 μM) were mixed with 1% of their fluorophore-labeled variants in 50 mM tris-HCl (pH 7.4), with 10% PEG and 20 μM ThT. Ten microliters was deposited on a Nunc glass base dish (Thermo Fisher Scientific) and imaged every 10 s from onset of condensation using a 20× objective on a Leica Stellaris 5 inverted confocal microscope.

### Microfluidics

#### Estimation of αSyn concentration for phase separation and aggregation in water-in-oil droplets

##### Fabrication of microfluidic devices

The fabrication of the microfluidic devices was performed on the basis of a previously established protocol (*49*, *52*, *53*). Briefly, a soft photolithographic process was used to fabricate the master from which microfluidic devices were made. A 50-μm photoresist (SU-8 3050) was spin coated onto a silicon wafer. This was soft baked at 95°C for 3 min. A film mask was placed on the wafer, and the whole system was irradiated with ultraviolet light to induce polymerization. The wafer was then baked at 95°C for 30 min. Last, the master was incubated in a solution of propylene glycol methyl ether acetate (Sigma-Aldrich) to aid in the development process. Elastomer polydimethylsiloxane (PDMS) was mixed with curing agent (Sylgard 184, Dow Corning) at a 10:1 ratio to fabricate the devices. This mixture was then incubated at 65°C and cured for a total of 3 hours. Once hardened, the PDMS was removed from the master, and holes were punched into the PDMS, serving as inlets and outlets. Last, the PDMS slab was attached to a glass slide by treatment with a plasma bonder (Diener Electronic).

##### Formation and confinement of water-in-oil droplets

Syringe pumps (Nemesys, CETONI) were used to control the flow rates within the microchannels. The aqueous phase was prepared by mixing αSyn with PEG as well as either 1% labeled αSyn or 20 µM ThT. At the junction, a solution of fluorinated oil (Fluorinert FC-40, Sigma-Aldrich) and 2% (w/w) fluorosurfactant (RAN Biotechnologies) intersected the aqueous phase leading to the formation of water-in-oil droplets. These water-in-oil droplets were directed toward an array of traps to maintain them into microfluidic confinement (*49*, *52*, *53*). The trapped water-in-oil droplets were then incubated at RT and allowed to shrink over time, resulting in an increase in the local αSyn and PEG concentrations, leading to αSyn phase separation and aggregation, which was monitored using fluorescence microscopy.

### Plate reader aggregation assays

To assess αSyn aggregation in the deposition pathway, αSyn splice isoforms were diluted to 10 μM in 50 mM tris-HCl (pH 7.4), with 50 μM ThT. The mixtures were transferred onto Corning 96-well Half-Area Black with Clear Flat Bottom Polystyrene Nonbinding Surface Microplates (Corning) with a 3-mm borosilicate bead (Merck) and incubated on a FLUOStar Omega plate reader (BMG Labtech) at 37°C. Aggregation traces were normalized, and half-times (*t*_1/2_) were calculated on the basis of the time when normalized fluorescence intensity reached 0.5. The experiments were performed with five repeats, and median traces were chosen for representation.
