# Supplementary material for: Aggregation of α-synuclein splice isoforms through a phase separation pathway
Source: Sci Adv. 2025 Apr 16;11(16):eadq5396. doi: 10.1126/sciadv.adq5396 (PMC12002138; doi:10.1126/sciadv.adq5396)
Supplement: Supplementary file 1 — Supplementary Methods Figs. S1 to S7 [file sciadv.adq5396_sm.pdf]

Science Advances
AAAS

Supplementary Materials for

Aggregation of α-synuclein splice isoforms through a phase separation pathway

Alexander Röntgen *et al.*

Corresponding author: Michele Vendruscolo, mv245@cam.ac.uk

Sci. Adv. **11**, eadq5396 (2025)
DOI: 10.1126/sciadv.adq5396

This PDF file includes:

Supplementary Methods
Figs. S1 to S7

Supplementary Methods

Transmission electron microscopy

αSyn isoforms (100 µM) were mixed with 10% PEG in 50 mM Tris-HCl, pH 7.4, and 5 µL were deposited on glow-discharged 3-mm 300-mesh carbon-film copper grids (EM Resolutions Ltd.). The sample was incubated for 5–8 min (αSyn-140–αSyn-98), blotted with Whatman filter paper and washed three times for 30 s with 5 µL MQ water. Finally, the sample was stained with 3 µL 2% (w/v) uranyl acetate for 40 s, blotted and air-dried. Images were acquired on a Talos F200X G2 transmission electron microscope (Thermo Fisher Scientific).

SDS-PAGE

NuPAGE LDS Sample Buffer (4x) was diluted into protein samples in a 1:4 ratio and the mixture was heated to 95 °C for 10 min. Samples were run on a 4–12% Bis-Tris NuPAGE gel with 1x NuPAGE SDS MES Running Buffer. SeeBlue Plus2 Pre-Stained Protein Standard (Thermo Fisher Scientific) served as protein marker. Subsequently, gels were stained using Instant Blue Coomassie Protein Stain (Abcam).

Fourier transform infrared (FTIR) spectroscopy

Aggregates of αSyn isoforms were washed three times at room temperature by centrifugation of the protein solution, discarding the supernatant and resuspending the pellet in MQ water. The aggregates were sonicated using a Sonopuls HD2070 (Bandelin) as required. FTIR spectra were acquired on a Vertex 70 FTIR spectrometer (Bruker) with a *DiamondATR* unit and a deuterated lanthanum α-alanine-doped triglycine sulphate detector. For all measurements conducted, an atmospheric and a carbon dioxide compensation was conducted by subtracting this from the FTIR spectra. To perform the experiment, 7.5 µL of the protein solution was deposited on the prism and spectra were acquired against a background of MQ water. Finally, the spectra were smoothened and normalised. Additionally, all measurements were performed at room temperature.

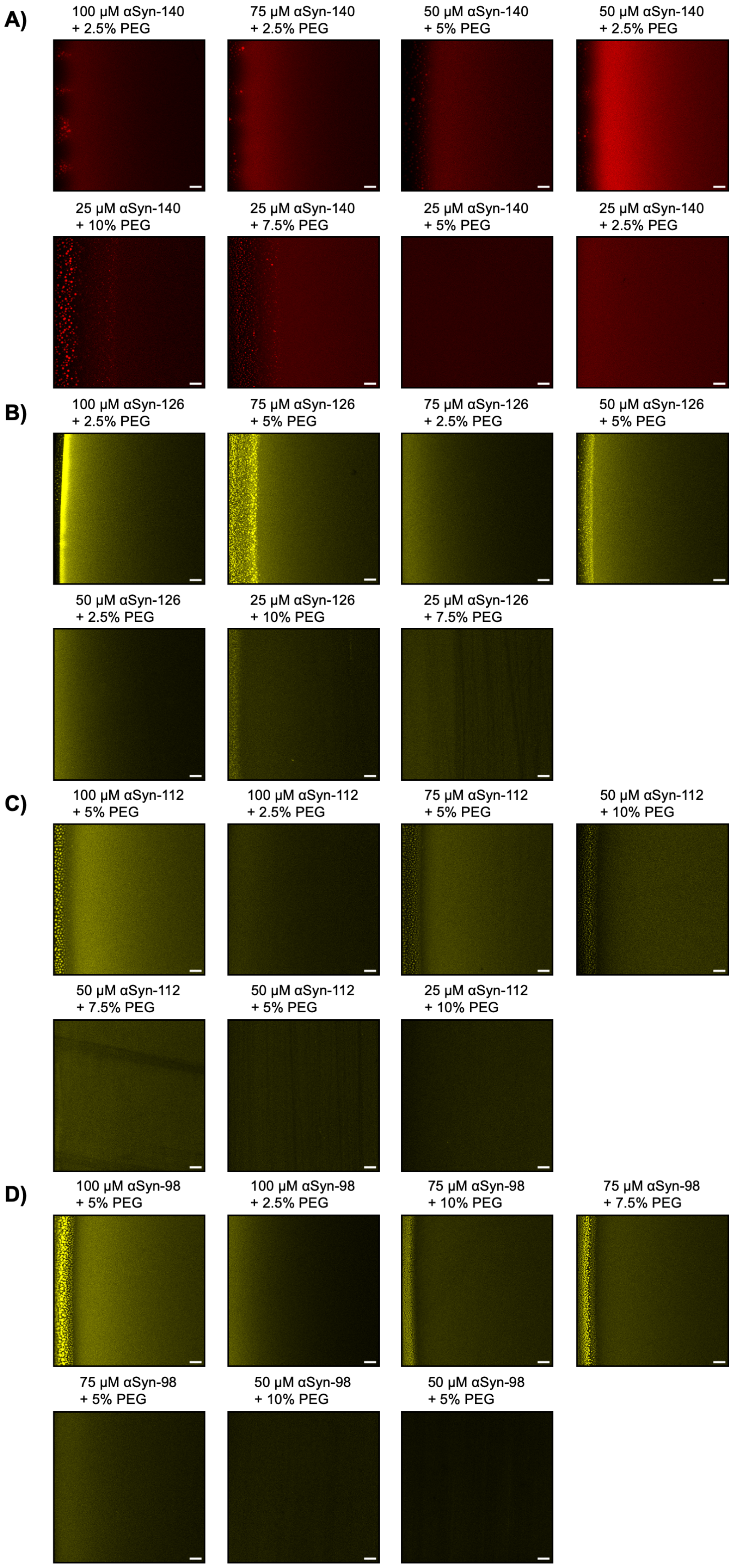

Supplementary Figure 1. Confocal microscopy images for all the LLPS phase boundary conditions tested of αSyn isoforms. (A) αSyn-140, **(B)** αSyn-126, **(C)** αSyn-112, **(D)** αSyn-98. Scale bar = 20 μm.

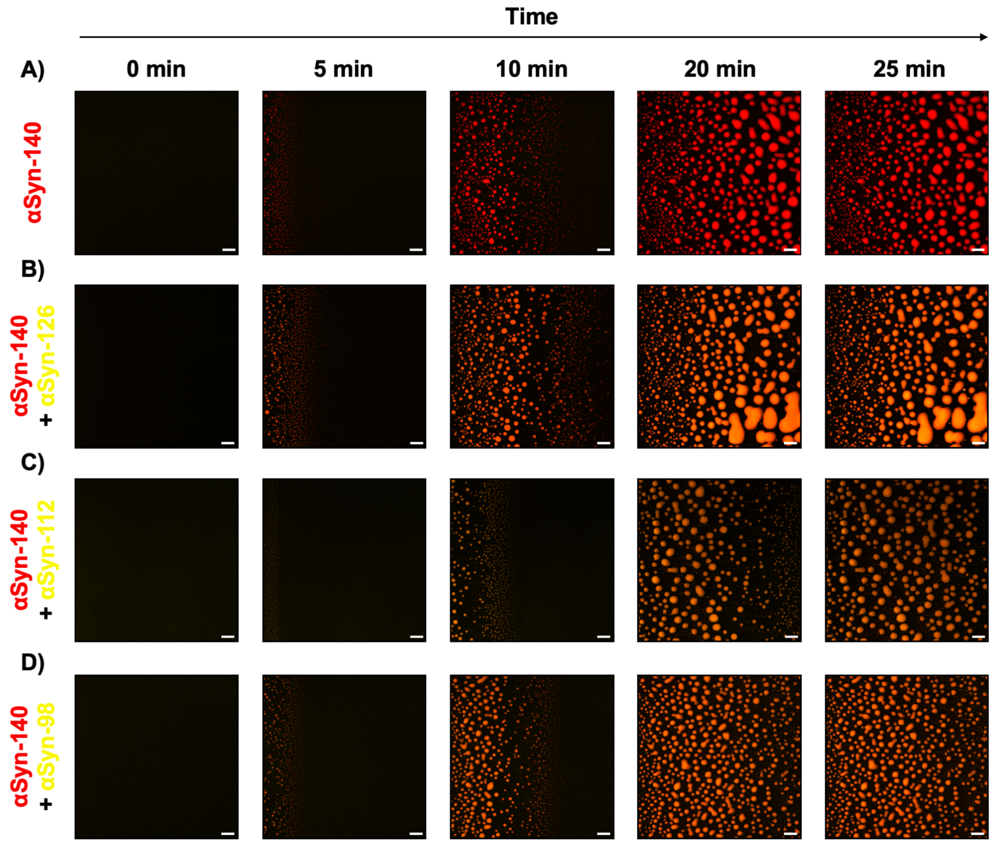

Supplementary Figure 2. Co-condensation of αSyn-140 with its alternative splice isoforms. Confocal microscopy images of the Alexa Fluor 647 channel (αSyn-140) were merged with images of the Alexa Fluor 555 channel (αSyn-126, αSyn-112, αSyn-98) to visualise the recruitment of the alternative αSyn splice isoforms into αSyn-140 condensates. **(A)** 100 μM αSyn-140, **(B)** 90 μM αSyn-140 + 10 μM αSyn-126, **(C)** 90 μM αSyn-140 + 10 μM αSyn-112, **(D)** 90 μM αSyn-140 + 10 μM αSyn-98 (1% fluorophore-labelled protein, respectively). Scale bar = 20 μm.

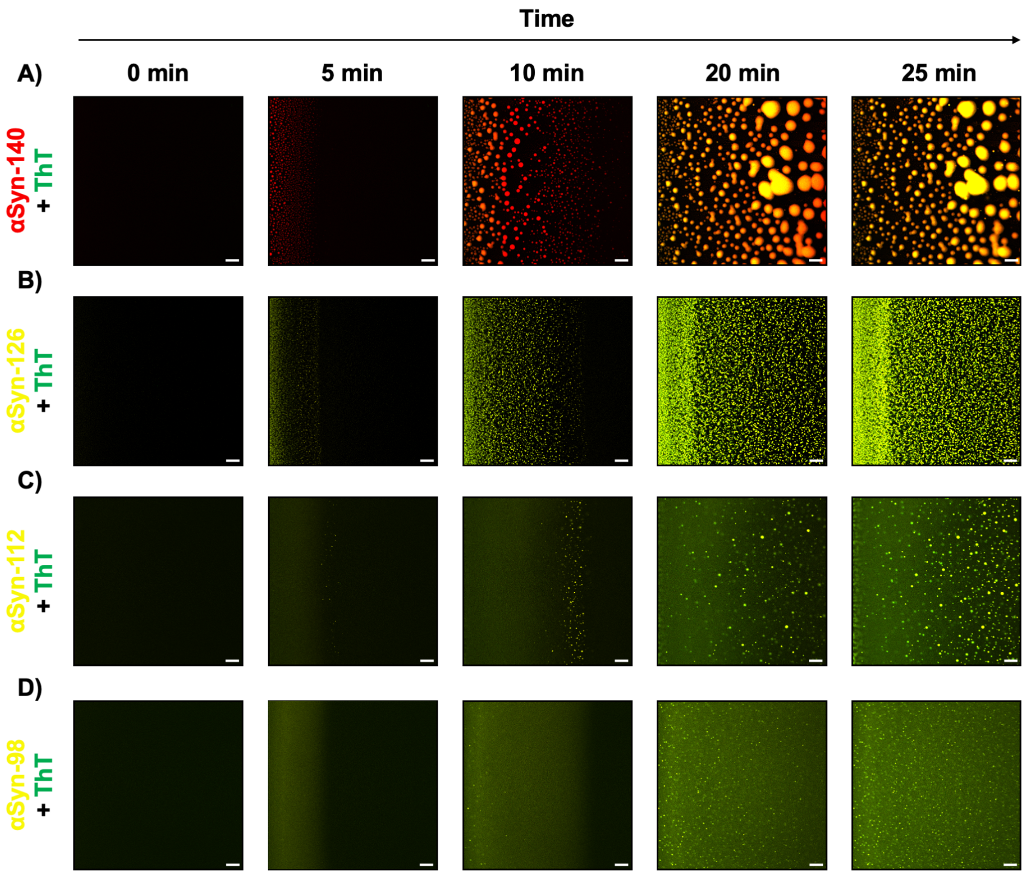

Supplementary Figure 3. Aggregation of αSyn isoforms within condensates. Confocal microscopy images of the **(A)** Alexa Fluor 647 (αSyn-140) or **(B-D)** Alexa Fluor 555 (αSyn-126, αSyn-112, αSyn-98) channels were merged with the ThT channel to visualise amyloid aggregates within αSyn condensates. Scale bar = 20 µm.

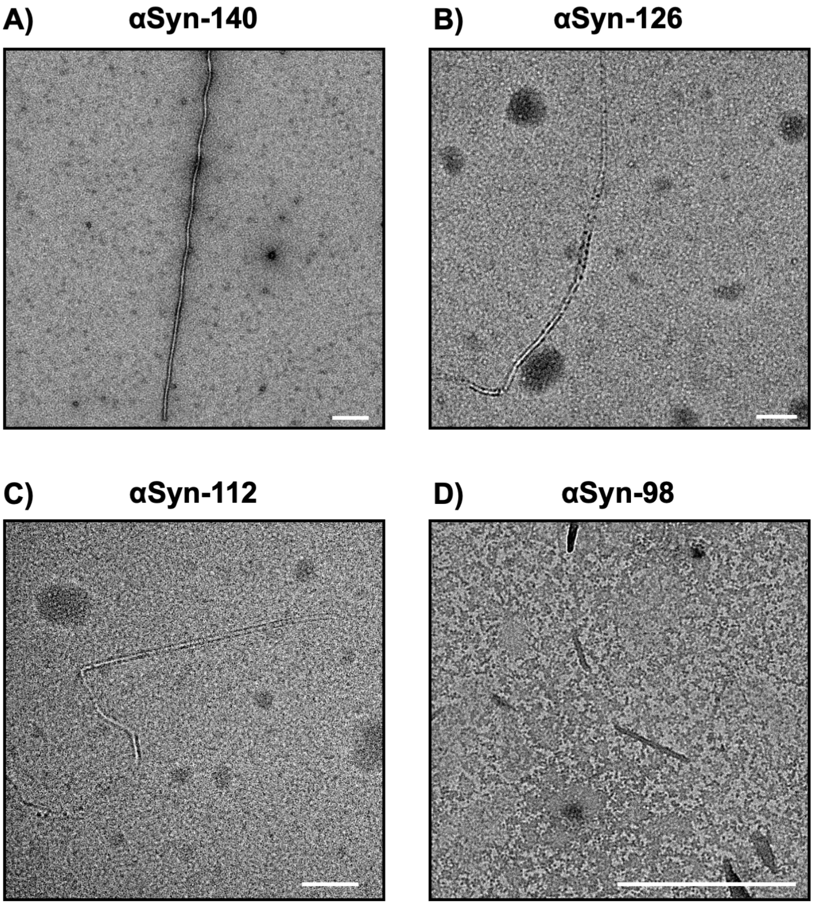

Supplementary Figure 4. TEM images of amyloid aggregates of αSyn splice isoforms following condensation and aggregation. (A) αSyn-140, **(B)** αSyn-126, **(C)** αSyn-112 and **(D)** αSyn-98. Scale bar = 200 nm.

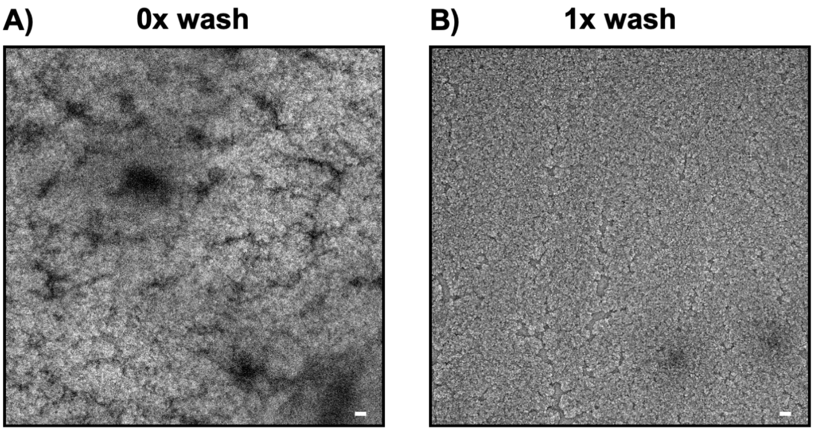

Supplementary Figure 5. TEM images of the condensation reaction with varying number of grid washes. (A) The grid was not washed. **(B)** The grid was washed once with 5 µL MQ water. Scale bar = 200 nm. In both cases, it is clear that multiple washing steps are required in order to observe αSyn fibrils.

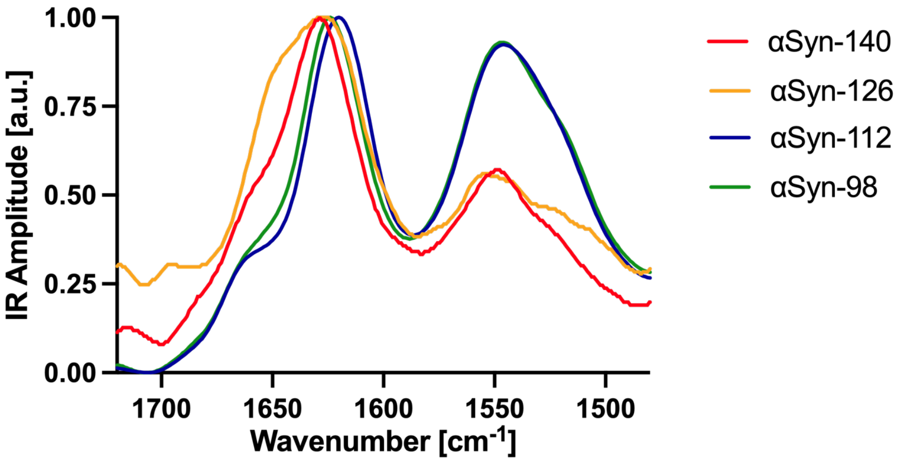

Supplementary Figure 6. FTIR spectra of αSyn isoform aggregates.

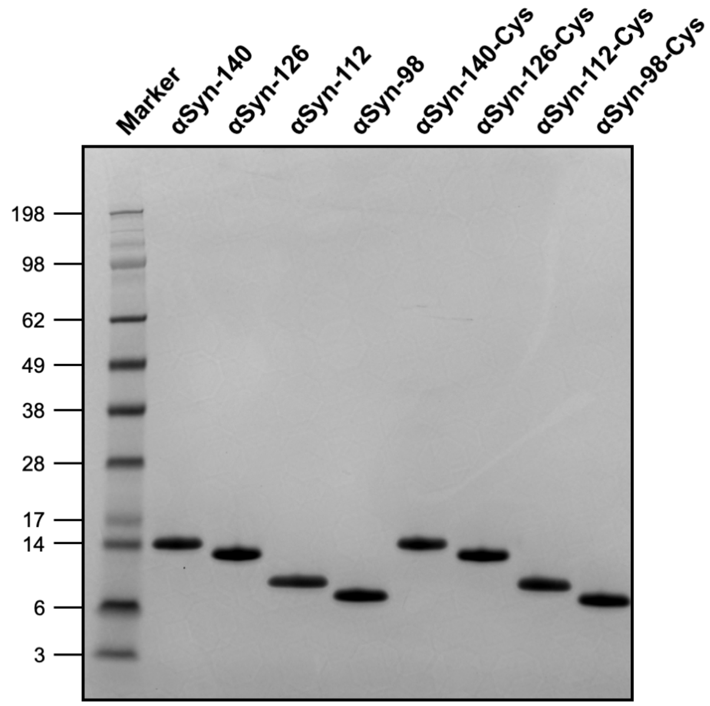

Supplementary Figure 7. SDS-PAGE of purified αSyn splice isoforms and cysteine mutants. The purity of the produced αSyn isoforms and their cysteine mutants was assessed by SDS-PAGE followed by Coomassie stain.
